# Potential impact of mepolizumab in stepping down anti-osteporotic treatment in corticosteroid-dependent asthma

**DOI:** 10.3389/fphar.2023.1183156

**Published:** 2023-05-09

**Authors:** Christian Domingo, Ana Sogo, Enrique Casado, Eva Martínez-Moragón, Marina Blanco-Aparicio, Teresa Carrillo, David Bañas-Conejero, María-Guadalupe Sánchez-Herrero

**Affiliations:** ^1^ Servicio de Neumología, Corporació Sanitària Parc Taulí, Barcelona, Spain; ^2^ Departamento de Medicina, Universitat Autònoma de Barcelona (UAB), Barcelona, Spain; ^3^ Servicio de Reumatología, Corporació Sanitària Parc Taulí, Barcelona, Spain; ^4^ Servicio de Neumología, Hospital Universitario Doctor Peset, Valencia, Spain; ^5^ Servicio de Neumología, Hospital Universitario A Coruña, A Coruña, Spain; ^6^ Servicio de Alergología, Hospital Universitario de Gran Canaria Doctor Negrín, Las Palmas de GranCanaria, Spain; ^7^ Departamento de Ciencias Médicas y Quirúrgicas, Universidad de Las Palmas Gran Canaria, Las Palmas de Gran Canaria, Spain; ^8^ Departamento Médico Specialty Care, GSK, Madrid, Spain

**Keywords:** asthma, mepolizumab, oral corticosteroid, osteoporosis treatment, anti-resorptive treatment

## Abstract

Oral corticosteroids (OCS) are commonly used for the acute management of severe asthma exacerbations or as maintenance therapy; however, chronic use is associated with significant toxicities, e.g., osteoporosis. In the REal worlD Effectiveness and Safety (REDES) study of mepolizumab in a multicentric Spanish cohort of asthma patients, mepolizumab effectively reduced clinically severe asthma exacerbations and decreased OCS dependence. This post-hoc analysis further evaluates mepolizumab’s de-escalation effect on OCS dose. Patients enrolled in REDES who had OCS consumption data available for 12 months pre- and post-mepolizumab treatment were included in this analysis. Primary outcomes were to determine the change in the proportion of patients eligible for anti-osteoporotic treatment due to the changes in OCS consumption before and after 1 year of mepolizumab treatment. All analyses are descriptive. Approximately one-third (98/318; 30.8%) of patients in REDES were on maintenance OCS at the time of mepolizumab treatment initiation. In REDES, mean cumulative OCS exposure decreased by 54.3% after 1 year of treatment. The proportion of patients on high-dose OCS (≥7.5 mg/day) fell from 57.1% at baseline to 28.9% after 12 months of mepolizumab treatment. Thus, 53.6% of OCS-dependent asthma patients treated with mepolizumab would cease to be candidates for anti-osteoporotic treatment according to guidelines thresholds.

## 1 Introduction

Corticosteroids (CS) have potent anti-inflammatory and immunosuppressant properties and are consequently used in a variety of indications in medicine. In asthma, they are widely used in an inhaled form. The oral (or parenteral) forms are principally used in the acute management of severe asthma exacerbations, or as alternative maintenance therapies when preferred treatments do not provide adequate disease control (Global Initiative for Asthma, 2021). However, chronic use of oral corticosteroids (OCS), including intermittent use at high doses (≥7.5 mg/day), is associated with significant toxicities due to the ubiquitous effects of OCS on organ systems. Toxicities include osteoporosis, fragility fractures, pneumonia, cardiac and cerebrovascular diseases, cataracts, sleep apnea, renal impairment, depression, anxiety, type 2 diabetes, and weight gain (Price et al., 2018).

Although an in-depth discussion of interventions to prevent systemic CS-related toxicities is beyond the scope of this article, the adverse skeletal effects of these agents deserve special mention because of the high prevalence of bone disorders in patients with severe asthma (Weare-Regales et al., 2021). Bone-related adverse effects are the result of OCS action in osteoblasts, osteocytes, and osteoclasts. After initiating OCS or systemic CS treatment, patients experience an initial bone resorption phase followed by stunted bone formation. This results in rapid deterioration of bone microarchitecture (bone quality), and a subsequent increase in fracture risk within the first 3–6 months of treatment, often before any bone mineral density (BMD) loss is detectable (Weare-Regales et al., 2021). Although it is dose dependent, the prevalence of fragility fractures in patients receiving CS has been estimated to be between 30% and 50% (Naranjo Hernández et al., 2019).

There is a need for increased clinician awareness and foresight regarding downstream toxicities caused by the management of asthma via CS. Effective disease management, avoidance of systemic CS-related osteoporotic fractures, and thus improvements in patient quality of life rely on early initiation of equally effective, OCS-sparing treatments, e.g., biologics.

Mepolizumab is a humanized monoclonal antibody marketed for T2 eosinophilic asthma, with a high affinity for interleukin-5 (IL-5), a key factor regulating the eosinophil pathway (involved in growth, differentiation, activation, recruitment, and survival processes) (Domingo Ribas et al., 2021). Mepolizumab has demonstrated effectiveness in reducing severe asthma exacerbations and improving lung function in the previously conducted REal worlD Effectiveness and Safety (REDES) study, a retrospective, real-world observational study of 318 patients with severe eosinophilic asthma in Spain (Domingo Ribas et al., 2021).

Here, we present a post-hoc analysis of the REDES study, which aimed to evaluate whether mepolizumab treatment de-escalated OCS dose, allowing for a subsequent discontinuation of anti-osteoporotic treatments in patients with severe eosinophilic asthma.

## 2 Methods

### 2.1 Study design and patients

Details of the REDES study design have been described previously (Domingo Ribas et al., 2021). Briefly, REDES (GSK ID: 213172) was a retrospective, real-world, phase IV, multicentre, observational cohort study that enrolled patients with severe eosinophilic asthma from across 24 Spanish hospitals. The observational period included 12 months pre- and post-mepolizumab treatment. Eligibility criteria for the REDES study included: patients ≥18 years of age with a clinical diagnosis of severe uncontrolled eosinophilic asthma who had initiated mepolizumab ≥12 months before the date of study inclusion and who had ≥12 months of relevant medical records prior to enrolment. The primary endpoint of the REDES study was the annual rate of clinically significant asthma exacerbations. Secondary endpoints included pre- and post-bronchodilator spirometric tests, changes in blood eosinophil counts, average OCS daily maintenance dose, and symptom control (assessed using the Asthma Control Test score) pre-to post-mepolizumab treatment. These results have been previously reported (Domingo Ribas et al., 2021).

The REDES study was performed in line with the guiding principles of the Declaration of Helsinki and order SAS/3470/2009. Approval was granted by the ethics committee of Hospital La Princesa, Madrid, Spain. Informed consent for the aggregation, anonymization and publication of health data was obtained from all participants included in the study. Separate ethics approval was not required for this analysis.

This post-hoc analysis of the REDES study evaluates the difference in the proportion of patients who would be candidates for anti-osteoporotic treatment (i.e., required ≥7.5 mg of prednisone per day), before and after 1 year of treatment with mepolizumab.

#### 2.1.1 Criteria for anti-osteoporotic treatment

**FIGURE 1:**
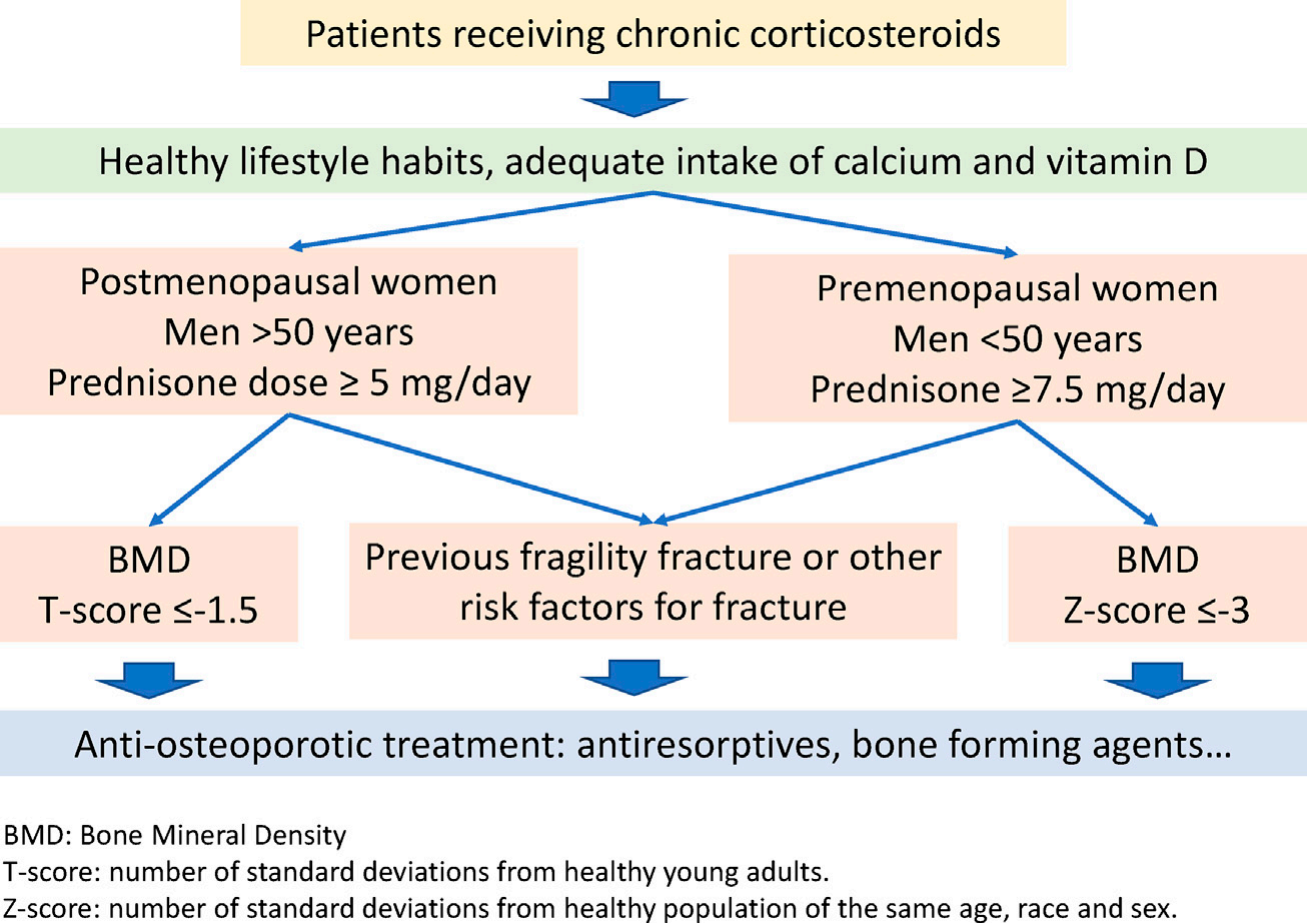
Recommendations to initiate preventive or anti-osteoporosis treatment in patients receiving glucocorticoids (Naranjo Hernández et al., 2019). BMD, bone mineral density; T-score: number of standard deviations from healthy young adults; Z-score: number of standard deviations from a healthy population of the same age, race, and sex.

To determine patient eligibility for initiation of anti-osteoporotic treatment (anti-resorptives or bone forming agents), this analysis uses the OCS thresholds provided by the Spanish Society of Rheumatology (Naranjo Hernández et al., 2019), which recommend initiating anti-osteoporotic treatment in individuals receiving more than 7.5 mg of prednisone per day (although this treatment can be initiated in patients receiving lower doses, depending on the age, BMD, previous fractures or other risk factors; Figure 1).

### 2.2 Post-hoc endpoints

The aim of this post-hoc analysis was to determine the change in the proportion of patients eligible for anti-osteoporotic treatment (according to the OCS daily dose defined in Section 2.1.1) through the evaluation of changes in OCS consumption before and after 1 year of mepolizumab treatment.

### 2.3 Statistical analysis

Continuous variables were described using the mean, median, standard deviation (SD), and interquartile range (IQR) values, while categorical variables were described using the number and percentage within categories. As all post-hoc analyses were descriptive, no statistical comparisons were performed.

## 3 Results

**FIGURE 2:**
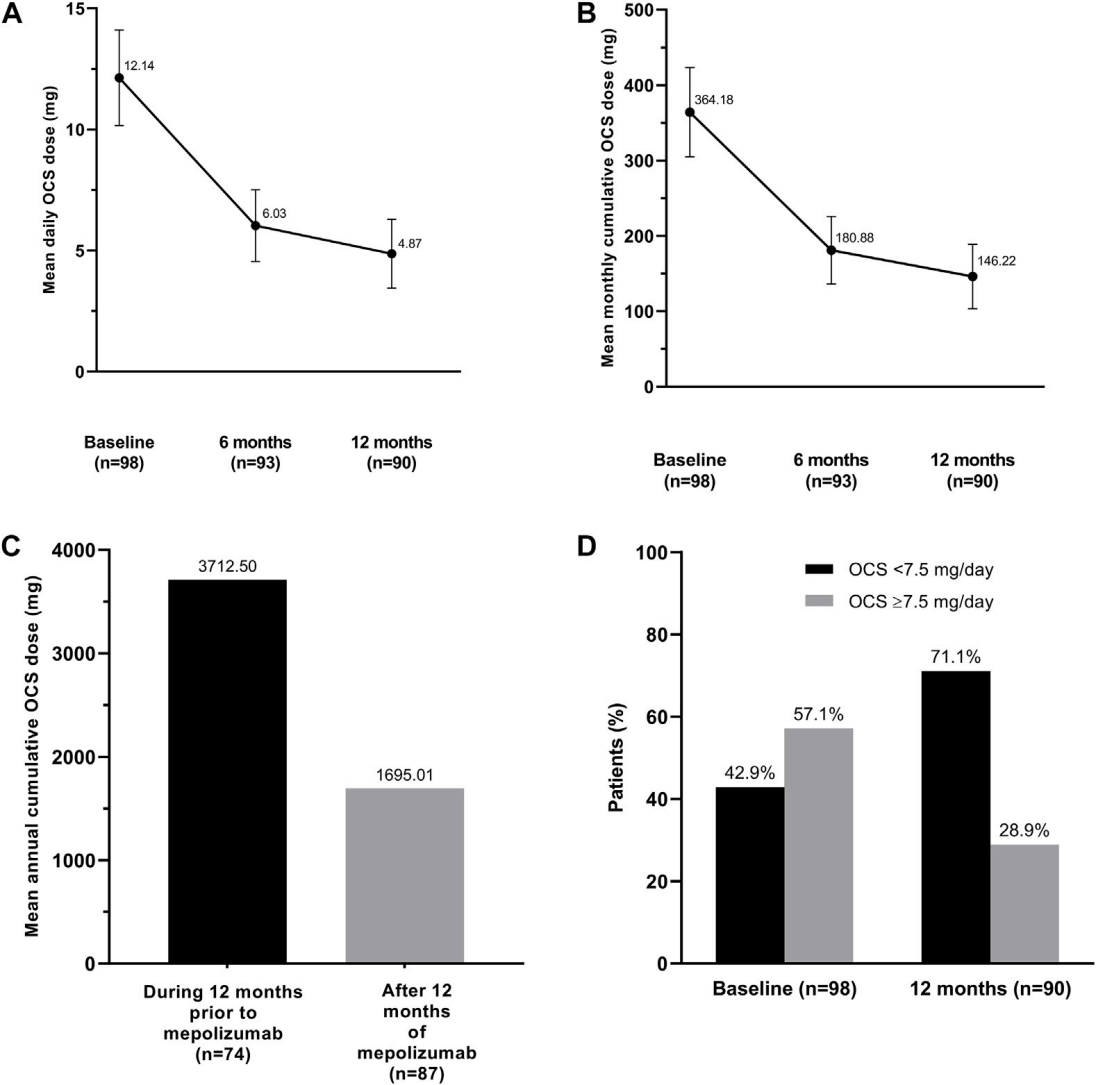
Changes in oral corticosteroid exposure (prednisolone equivalents): **(A)** mean daily dose and **(B)** mean monthly cumulative dose during mepolizumab treatment, **(C)** annual cumulative dose during the year prior to mepolizumab and after 12 months of receiving mepolizumab treatment, and **(D)** proportion of patients receiving <7.5 mg/day or ≥7.5 mg/day at entry and after 1 year of mepolizumab. OCS, oral corticosteroids. The error bars represent standard error.

In the REDES study, almost one-third of patients (98/318; 30.8%) were on maintenance OCS at the time of initiating mepolizumab treatment; mean (SD) OCS dose was 12.14 (9.97) mg/day. Figure 2 shows changes in the patients’ OCS exposure during the study. Cumulative OCS exposure was 54.3% lower after 1 year of mepolizumab treatment than in the year prior to starting mepolizumab (mean [SD] cumulative dose was reduced to 1695.01 [2262.08] mg, from 3712.50 [2983.36] mg; Figure 2C). Of the 98 patients on OCS, 47.8% discontinued CS treatment by 12 months (Domingo Ribas et al., 2021).

Importantly, the number of patients on high-dose OCS (≥7.5 mg/day) fell from 56/98 (57.1%) at baseline to 26/90 (28.9%) after 12 months of mepolizumab treatment (Figure 2D). Thus, 30 (53.6%) CS-dependent asthma patients treated with mepolizumab in the REDES study would cease to be candidates for anti-osteoporotic treatment according to guidelines thresholds. Theoretically, anti-osteoporotic treatments could have also been withdrawn from these patients, reducing the risk of long-term drug-related complications.

At 12 months, nine patients (2.8%) reported 13 adverse events while on treatment with mepolizumab, none of which were severe. The complete list of adverse events, which were generally mild and temporary, has been previously published (Domingo Ribas et al., 2021).

## 4 Discussion

As OCS or parenteral CS therapies are likely to remain as a tool used for the management of acute severe asthma exacerbations for the foreseeable future, it is important to consider the impact of episodic CS courses on the risk of CS-related toxicities. Episodic systemic CS courses increase CS exposure beyond that of maintenance oral/inhaled CS. The risk of osteoporosis diagnosis and fracture has been shown to increase per 1-g increase in systemic CS exposure in patients with asthma (Price et al., 2018). In recognition of this risk, anti-osteoporotic treatment is recommended in adults with high cumulative CS exposure (>5 g/year) (Buckley et al., 2017; Naranjo Hernández et al., 2019). Moreover, a recent analysis found large inter-person variability in the risk of oral CS-related toxicity among patients with severe asthma, as measured by the Glucocorticoid Toxicity Index (GTI), with only a modest correlation between recent OCS exposure and GTI score (McDowell et al., 2021). In contrast, age and disease-specific quality-of-life scores correlated more strongly with the GTI score, likely reflecting the impact of lifetime OCS exposure.

Compared to CS, asthma biologics such as mepolizumab have shown a more favorable safety profile, which, together with the efficacy of biologics, has helped position CS as a last resort choice after biologics. In the clinical studies of mepolizumab, the most commonly reported adverse drug reactions were headache, injection site reactions, and back pain, up to 4.8 years after treatment initiation (Khurana et al., 2019; Mitchell and Leigh, 2019).

Although chronic use of prednisolone-equivalent OCS doses as low as 2.5 mg/day are enough to increase the risk of fractures (Zeiger et al., 2017), this risk is significantly higher in individuals taking ≥7.5 mg/day (van Staa et al., 2000). Consequently, osteoporosis prevention treatment is recommended for individuals (with additional risk factors) who are aged <40–50 years receiving prednisolone >7.5 mg/day (Buckley et al., 2017; Naranjo Hernández et al., 2019) or aged >50 years receiving ≥5 mg/day (Naranjo Hernández et al., 2019).

The minimization or avoidance of chronic OCS use in asthma treatment is pertinent in reducing osteoporosis and fracture risk and lessening patient exposure to other pharmacologic interventions. In this respect, the data derived from the REDES study support the benefits mepolizumab may offer in decreasing the indication of anti-osteoporotic treatment.

Despite asthma guidelines recommending mepolizumab and other biologics over OCS as maintenance therapies for severe asthma (Global Initiative for Asthma, 2021), it is important to note that biologics do not completely eliminate the need for OCS in all patients, nor do they definitively prevent exacerbations. Physicians managing severe asthma should continue to remain vigilant in assessing CS-related toxicity risk in patients receiving biologics or long-term OCS, particularly in patients carrying additional osteoporotic risk factors (Weare-Regales et al., 2021).

This post-hoc analysis shows that mepolizumab allows the reduction of OCS in CS-dependent asthma patients, lowering their fracture risk and therefore allowing them to discontinue anti-osteoporotic treatment, avoiding or reducing potential long-term drug-related complications, such as atypical femoral fractures or osteonecrosis of the jaw.

The REDES study and, therefore, this post-hoc analysis have some limitations. The most important limitation is that our calculation of the proportion of patients who were candidates for anti-osteoporotic treatment discontinuation is only an estimation. Another limitation is that although participating centers had dedicated asthma units for data collection, the retrospective nature of the study may have resulted in incomplete data collection and/or data may have been subject to time-varying confounders. An example of this is the lack of reliable data on cumulative OCS dose that included all OCS courses patients had received during the study, meaning we had to limit our analysis to those patients receiving a maintenance daily OCS dose.

## 5 Conclusion

Mepolizumab has shown a de-escalation effect in OCS maintenance dose, as well as a reduction in cumulative CS exposure, in a high proportion of severe asthma patients. However, its potential effect on anti-osteoporotic treatment had not been reported. Our study quantifies for the first time that 53.6% of CS-dependent severe asthma patients, would cease to be candidates for anti-osteoporotic treatment after 12 months of mepolizumab therapy.

## Data Availability

The original contributions presented in the study are included in the article/Supplementary Material, further inquiries can be directed to the corresponding author: Christian Domingo, cdomingo@tauli.cat.

## Abbreviations

ACT, asthma control test; CS, corticosteroids; GTI, Glucocorticoid Toxicity Index; IL-5, interleukin-5; IQR, interquartile range; OCS, oral corticosteroids; REDES, REal worlD Effectiveness and Safety Study; SD, standard deviation.
